# Supplementary material for: Identification of photoperiod-induced specific miRNAs in the adrenal glands of Sunite sheep (Ovis aries)
Source: Front Vet Sci. 2022 Jul 22;9:888207. doi: 10.3389/fvets.2022.888207 (PMC9354845; doi:10.3389/fvets.2022.888207)
Supplement: Supplementary file 1 [file Data_Sheet_1.ZIP › Supplementary file/miRDeep2 file.zip!/miRDeep2 file/Novel_Structure/SPLP42A2/Novel_501.pdf]

Provisional ID	: NC_019467.2_21876
Score total	: 1.4
Score for star read(s)	: -1.3
Score for read counts	: 0
Score for mfe	: 1.1
Score for randfold	: 1.6
Score for cons. seed	:
Total read count	: 22
Mature read count	: 22
Loop read count	: 0
Star read count	: 0

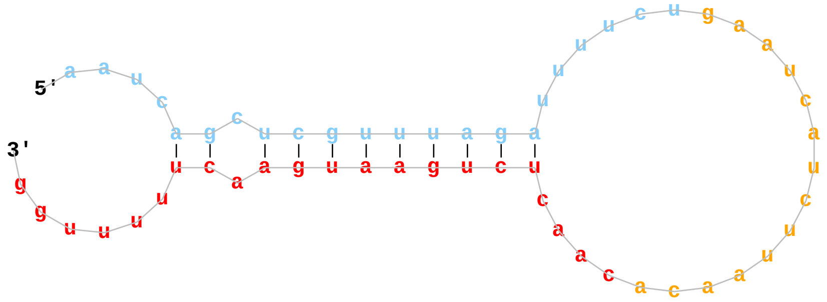

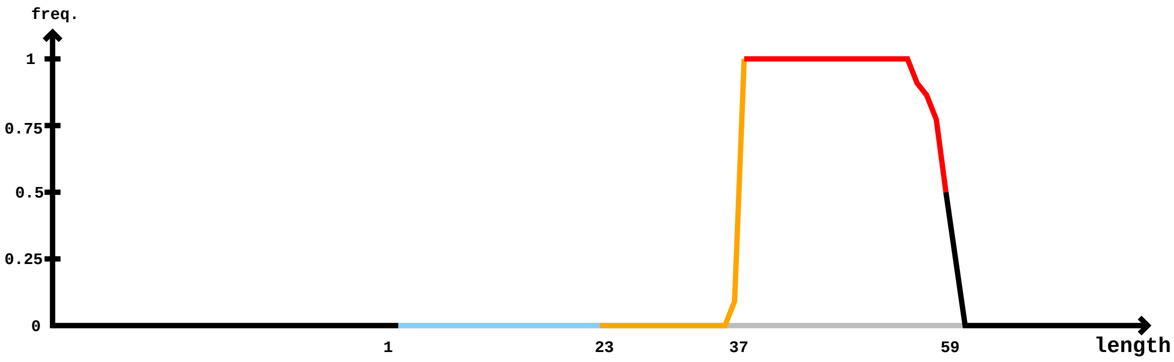

Star Mature

```
5'- uucugagcuugguauuagugauauuggcuuggcuaaucagcucguuuagauuuucugaaucaucuuaacacaacucugaaugaacuuuuuggccagcccaaauacaagcuca   -3'    exp
    ...((((((((.((((..((.....((((.((((((..((.(((((((((........................))))))))).))..))))))))))))))))))))))))   reads      mm        sample
    .....................................................................aAaacucugaaugaacuuu........................   2          1         seq
    ......................................................................Aaacucugaaugaacuuuu.......................   1          1         seq
    ......................................................................caacucugaaugaacuuuuu......................   2          0         seq
    ......................................................................caacucugaaugaacuuuuug.....................   2          0         seq
    ......................................................................Aaacucugaaugaacuuuuug.....................   4          1         seq
    ......................................................................caacucugaaugaacuuuuugg....................   3          0         seq
    ......................................................................Aaacucugaaugaacuuuuugg....................   8          1         seq
```
